# Peristaltic Transport of Prandtl-Eyring Liquid in a Convectively Heated Curved Channel

**DOI:** 10.1371/journal.pone.0156995

**Published:** 2016-06-15

**Authors:** Tasawar Hayat, Shahida Bibi, Fuad Alsaadi, Maimona Rafiq

**Affiliations:** 1 Department of Mathematics, Quaid-i-Azam University 45320, Islamabad 44000, Pakistan; 2 Department of Electrical and Computer Engineering, Faculty of Engineering, King Abdulaziz University, Jeddah 21589, Saudi Arabia; North China Electric Power University, CHINA

## Abstract

Here peristaltic activity for flow of a Prandtl-Eyring material is modeled and analyzed for curved geometry. Heat transfer analysis is studied using more generalized convective conditions. The channel walls satisfy complaint walls properties. Viscous dissipation in the thermal equation accounted. Unlike the previous studies is for uniform magnetic field on this topic, the radial applied magnetic field has been utilized in the problems development. Solutions for stream function (*ψ*), velocity (*u*), and temperature (θ) for small parameter β have been derived. The salient features of heat transfer coefficient Z and trapping are also discussed for various parameters of interest including magnetic field, curvature, material parameters of fluid, Brinkman, Biot and compliant wall properties. Main observations of present communication have been included in the conclusion section.

## Introduction

Peristaltic transport holds a considerable position in physiology and engineering. Extensive research has been addressed under different situations since the seminal works of Latham [1] and Shapiro et al. [2]. Locomotion of worm, gliding movement of some bacteria, corrosive and sanitary liquids transport, heart lung machine and roller and finger pumps also uses this mechanism for their working. Heat transfer in peristalsis is further significant in chemical and pharmaceutical industries, hemodialysis, oxygenation, tissue analysis, thermotherapy and human thermoregulatory process. Heat transfer is also quite prevalent in several peristaltic pumps. Gul et al. [3] discussed the effect of temperature dependent viscosity on the flow of third grade fluid over vertical belt. Gul et al. [4, 5] also study the heat transfer analysis by considering oscillating vertical and inclined belt. Besides this, advancement is also made about the interaction of magnetohydrodynamics in peristalsis, which finds great importance in connection with certain problems for motion of conductive fluids in physiology, for instance, the blood and blood pumps machines, hyperthermia, cancer therapy, drug delivery transport, magnetic resonance imaging (MRI) and theoretical research about operation of peristaltic magnetohydrodynamic (MHD) compressors. Motivated by all the aforementioned facts the recent investigators are also engaged in the analysis of peristalsis through diverse aspects. Few recent studies and several interesting references in this direction can be seen in the attempts [6–21].

All the aforementioned attempts and existing information on this topic witness that much attention has been given to the flows with peristalsis in a planer channel which seems inadequate in reality. It is because of the fact that most of the ducts in physiological and industrial applications are curved. Influence curvature on peristaltic transport liquid is discussed in only some studies. Sato et al. [22] initially examined the curvature effect in the peristalsis of viscous fluid. Ali et al. [23] reconsidered the problem of ref [24] in wave frame. Hayat et al. [25] study the peristaltic phenomenon for viscous fluid in curved geometry. The peristaltic flow of third order and Carreau-Yasuda materials in curved flow configuration has been discussed by Ali et al. [26] and Abbasi et al. [27] respectively. Heat/mass transport in peristalsis of pseudoplastic, Johnson-Segalman and third grade fluids are explored by Hina et al. [28–29] and Hayat et al. [30]. On the other hand it has also been noted that heat transfer in previous studies related to peristalsis has been dealt with either prescribing temperature or heat flux at the channel walls. Scarce information is available for peristalsis involving heat transfer through convective conditions (see [31–33]).

The facts of present attempt is to advance the theory of peristalsis of non-Newtonian materials via three important aspects i.e. curved channel, convective heat transfer condition and radial magnetic field. Hence we model here the governing flow problem employing constitutive relations of Prandtl-Eyring fluid. The flow formulation is completed through compliant properties of channel walls. Arising nonlinear analysis is computed for the series solutions. Arrangement of paper is as follows. Next section formulates the problems for flow and temperature. Section three includes solution expressions for the stream function, temperature and heat transfer coefficient. Discussion to graph of different physical quantities for various parameters is assigned in section four. section five consists of conclusions.

## Formulation

**Fig 1:**
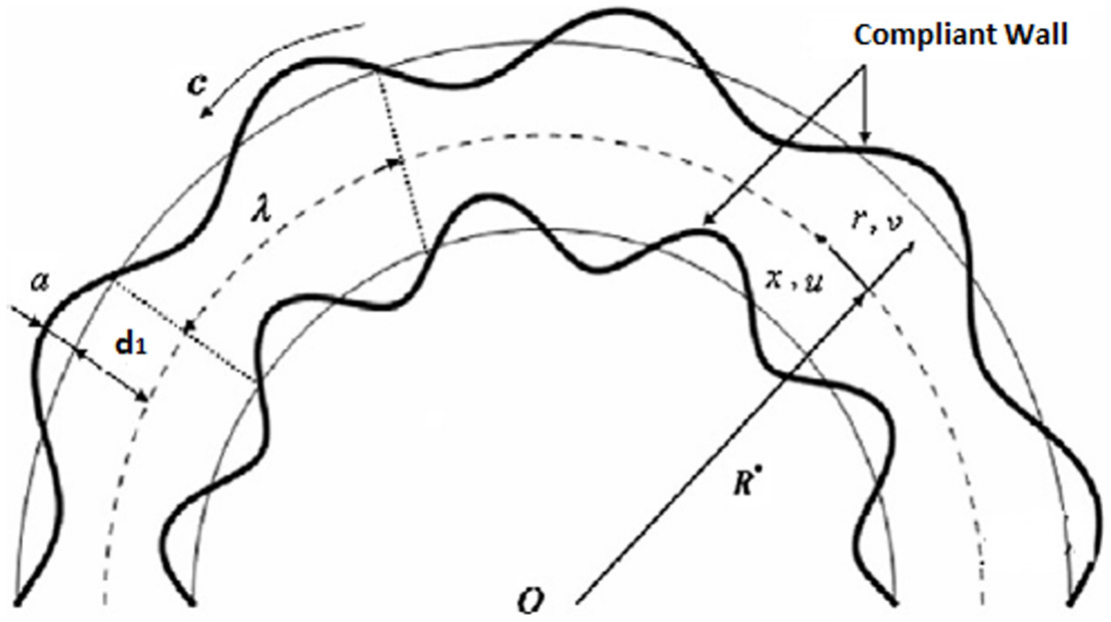
Geometry of the problem.

Consider a channel in curved shape with width 2*d*_*1*_ looped in a circle with radius R* and center *O* (see Fig 1). Coordinate axis are selected in such a way that x-axis lies along the length of the channel and r-axis lies normal to it. An incompressible electrically conducting Prandtl-Eyring fluid fills the channel. Flow in the channel is generated by propagating peristaltic waves along the channel walls in the axial direction with constant speed *c*. A radial magnetic field $\text{B }=(\frac{B_{0}}{r+R^{*}}\;,0,0)$ is applied. Induced magnetic field is neglected for small magnetic Reynolds number assumption. Electric field is further absent.

The wave form at the walls are

$$r=\pm \eta \left(\text{x},\text{t}\right)=\pm \left[{\text{d}}_{1}+\text{asin}\left(\frac{2\pi }{\lambda }\left(\text{x }–\text{ ct}\right)\right)\right],$$ (1)

here *a* depicts the wave amplitude, λ the wavelength and t the time. The constitutive equations are:

$$\frac{\partial \left[\left(r+R^{*}\right)v\right]}{\partial r}+R^{*}\frac{\partial u}{\partial x}=0,$$ (2)

$$\rho \left(\frac{\partial v}{\partial t}+v\frac{\partial v}{\partial r}+\frac{R^{*}u}{r+R^{*}}\frac{\partial v}{\partial x}-\frac{u^{2}}{r+R^{*}}\right)=-\frac{\partial p}{\partial r}+\frac{1}{r+R^{*}}\frac{\partial \left[\left(r+R^{*}\right)S_{rr}\right]}{\partial r}+\frac{R^{*}}{r+R^{*}}\frac{\partial S_{xr}}{\partial x}-\frac{S_{xx}}{r+R^{*}}-\frac{\sigma vB_{0}^{2}}{{\left(r+R^{*}\right)}^{2}},$$ (3)

$$\rho \left(\frac{\partial u}{\partial t}+v\frac{\partial u}{\partial r}+\frac{R^{*}u}{r+R^{*}}\frac{\partial u}{\partial x}+\frac{uv}{r+R^{*}}\right)=-\frac{R^{*}u}{r+R^{*}}\frac{\partial p}{\partial x}+\frac{1}{{\left(r+R^{*}\right)}^{2}}\frac{\partial \left[{\left(r+R^{*}\right)}^{2}S_{rx}\right]}{\partial r}+\frac{R^{*}}{r+R^{*}}\frac{\partial S_{xx}}{\partial x}-\frac{\sigma uB_{0}^{2}}{{\left(r+R^{*}\right)}^{2}},$$ (4)

$$\rho C_{p}\left(\frac{\partial T}{\partial t}+v\frac{\partial T}{\partial r}+\frac{R^{*}u}{r+R^{*}}\frac{\partial T}{\partial x}\right)=\kappa \left(\frac{\partial^{2}T}{\partial r^{2}}+\frac{1}{r+R^{*}}\frac{\partial T}{\partial r}-{\left(\frac{R^{*}}{r+R^{*}}\right)}^{2}\frac{\partial^{2}T}{\partial x^{2}}\right)+(S_{rr}-S_{xx})\left(\frac{\partial v}{\partial r}\right)+S_{xr}\left(\frac{\partial u}{\partial r}+\frac{R^{*}}{r+R^{*}}\frac{\partial v}{\partial x}-\frac{u}{r+R^{*}}\right),$$ (5)

where *σ* is the electrical conductivity of fluid, *S*_*ij*_ (*i*, *j* = *r*, *x*) the components of extra stress tensor, *C*_*p*_ denotes the specific heat, *ρ* the density, *κ* stands for thermal conductivity and *T* the temperature of fluid.

Extra stress tensor **(S)** for Prandtl-Eyring fluid is written as:

$$S=\frac{1}{\dot{\gamma }}Asinh^{-1}\left(\frac{\dot{\gamma }}{C}\right)A_{1},$$ (6)

$$\dot{\gamma }=\sqrt{\frac{1}{2}\Pi },$$ (7)

$$\Pi =tr{\left(A_{1}\right)}^{2},$$

$$A_{1}=L+L^{transpose}\;,$$

where **L** = (grad **V**) and *A*/*C* are material constants of Prandtl-Eyring fluid model.

The boundary conditions have been assumed in the form

u=0 at r=±η (8)

$$k\frac{\partial T}{\partial r}=-h_{1}\left(T-T_{0}\right)\;at\;r=+\eta ,$$ (9)

$$k\frac{\partial T}{\partial r}=-h_{1}\left(T_{0}-T\right)\;at\;r=-\eta ,$$ (10)

$$R^{*}\left[-\tau \frac{\partial^{3}\eta }{\partial x^{3}}+{\text{m}}_{1}\frac{\partial^{3}\eta }{\partial x\partial t^{2}}+\text{d}\frac{\partial^{2}\eta }{\partial t\partial x}\right]=\frac{1}{\left(r+R^{*}\right)}\frac{\partial \left[{\left(r+R^{*}\right)}^{2}S_{rx}\right]}{\partial r}+\frac{\partial S_{xx}}{\partial x}-\rho \left(r+R^{*}\right)\left(\frac{\partial u}{\partial t}+v\frac{\partial u}{\partial r}+\frac{R^{*}u}{r+R^{*}}\frac{\partial u}{\partial x}+\frac{uv}{r+R^{*}}\right)-\frac{\sigma uB_{0}^{2}}{{\left(r+R^{*}\right)}^{2}}\;at\;r=\pm \eta .$$ (11)

Here *T*_*0*_ is the temperature at both upper and lower walls of the channel, *h*_*1*_ the heat transfer coefficient at upper/lower walls whereas *S*_*xx*_, *S*_*rr*_, *S*_*rx*_ and *S*_*xr*_ are the elements of **S**. Moreover

$$L=-\tau \frac{\partial^{2}}{\partial x^{2}}+{\text{m}}_{1}\frac{\partial^{2}}{\partial t^{2}}+\text{d}\frac{\partial }{\partial t},$$ (12)

where *m*_*1*_, *τ* and *d* are the mass per unit area, longitudinal tension and the viscous damping coefficient respectively. Having

$$x^{*}=\frac{x}{\lambda },\;r^{*}=\frac{r}{d_{1}},\;p^{*}=\frac{d_{1}^{2}p}{c\mu \lambda },t^{*}=\frac{ct}{\lambda },\;u^{*}=\frac{u}{c},\;v^{*}=\frac{v}{c},$$

$$S^{*}=\frac{dS}{\mu c},h=\frac{\eta }{d_{1}},\text{K}=\frac{R^{*}}{d_{1}},\delta =\frac{d_{1}}{\lambda },\theta =\frac{T-T_{0}}{T_{0}}.$$

Eqs (3)–(11) can be reduced as follows:

$$Re\delta \left[\delta \frac{\partial v}{\partial t}+v\frac{\partial v}{\partial r}+\frac{\text{K}\delta u}{\left(r+\text{K}\right)}\frac{\partial v}{\partial x}-\frac{u^{2}}{\left(r+\text{K}\right)}\right]=-\frac{\partial p}{\partial r}+\delta \left[\frac{1}{\left(r+\text{K}\right)}\frac{\partial }{\partial r}\left[\left(r+\text{K}\right)S_{rr}\right]+\frac{\text{K}\delta }{\left(r+\text{K}\right)}\frac{\partial }{\partial x}S_{xr}-\frac{S_{xx}}{\left(r+\text{K}\right)}\right],$$ (13)

$$-Re\left[\delta \frac{\partial u}{\partial t}+v\frac{\partial u}{\partial r}+\frac{\text{K}\delta u}{\left(r+\text{K}\right)}\frac{\partial u}{\partial x}+\frac{uv}{\left(r+\text{K}\right)}\right]=-\frac{\text{K}}{\left(r+\text{K}\right)}\frac{\partial p}{\partial x}+\frac{1}{{\left(r+\text{K}\right)}^{2}}\frac{\partial }{\partial r}\left[{\left(r+\text{K}\right)}^{2}S_{rx}\right]+\frac{\text{K}\delta }{\left(r+\text{K}\right)}\frac{\partial }{\partial x}S_{xx}-\frac{H^{2}u}{{\left(r+\text{K}\right)}^{2}}\;,$$ (14)

$$Re\left[\delta \frac{\partial }{\partial t}+v\frac{\partial }{\partial r}+\frac{\text{K}\delta u}{\left(r+\text{K}\right)}\frac{\partial }{\partial x}\right]\theta =\frac{1}{Pr}\left[\frac{\partial^{2}}{\partial r^{2}}+\frac{1}{\left(r+\text{K}\right)}\frac{\partial }{\partial r}+\delta^{2}\frac{\partial^{2}}{\partial x^{2}}\right]\theta +Ec\left[\left(S_{rr}-S_{xx}\right)\frac{\partial v}{\partial r}+S_{xr}\left(\frac{\partial u}{\partial r}+\frac{\text{K}\delta }{\left(r+\text{K}\right)}\frac{\partial v}{\partial x}-\frac{u}{\left(r+\text{K}\right)}\right)\right],$$ (15)

with the following non-dimensional boundary conditions

u=0 at r=±h, (16)

$$\frac{\partial \theta }{\partial r}+Bi_{1}\theta =0\;at\;r=h,$$ (17)

$$\frac{\partial \theta }{\partial r}-Bi_{1}\theta =0\;at\;r=-h,$$ (18)

$$\left[E_{1}\frac{\partial^{3}h}{\partial x^{3}}+E_{2}\frac{\partial^{3}h}{\partial x\partial t^{2}}+E_{3}\frac{\partial^{2}h}{\partial t\partial x}\right]=-\frac{Re\left(r+\text{K}\right)}{K}\left[\delta \frac{\partial u}{\partial t}+v\frac{\partial u}{\partial r}+\frac{\text{K}\delta }{\left(r+\text{K}\right)}\frac{\partial u}{\partial x}+\frac{uv}{\left(r+\text{K}\right)}\right]+\frac{1}{\text{K}\left(r+\text{K}\right)}\frac{\partial }{\partial r}\left[{\left(r+\text{K}\right)}^{2}S_{rx}\right]+\;\frac{\delta }{\text{K}}\frac{\partial S_{xx}}{\partial x}-\frac{H^{2}u}{\text{K}{\left(r+\text{K}\right)}^{2}}\;at\;r=\pm h,$$ (19)

with

h=±[1+ϵsin2π(x−t)].

Taking the stream function *ψ (x*, *r*, *t)* by

$$u=-\frac{\partial \psi }{\partial r}\;,v=\delta \frac{\text{K}}{\left(r+\text{K}\right)}\frac{\partial \psi }{\partial x}.$$ (20)

Eq (2) is identically satisfied and Eqs (13)–(19) after utilizing lubrication approximation take the forms:

$$\frac{\partial p}{\partial r}=0,$$ (21)

$$-\frac{\text{K}}{\left(r+\text{K}\right)}\frac{\partial p}{\partial x}+\frac{1}{{\left(r+\text{K}\right)}^{2}}\frac{\partial }{\partial r}\left[{\left(r+\text{K}\right)}^{2}S_{rx}\right]+\frac{H^{2}\psi_{r}}{{\left(r+\text{K}\right)}^{2}}=0,$$ (22)

$$\left[\frac{\partial^{2}}{\partial r^{2}}+\frac{1}{\left(r+\text{K}\right)}\frac{\partial }{\partial r}\right]\theta =-Br\left[S_{xr}\left(-\psi_{rr}+\frac{\psi_{r}}{\left(r+\text{K}\right)}\right)\right],$$ (23)

$$\psi_{r}=0\;at\;r=\pm h,$$ (24)

$$\frac{\partial \theta }{\partial r}+Bi_{1}\theta =0\;at\;r=h,$$ (25)

$$\frac{\partial \theta }{\partial r}-Bi_{1}\theta =0\;at\;r=-h,$$ (26)

$$\left[E_{1}\frac{\partial^{3}}{\partial x^{3}}+E_{2}\frac{\partial^{3}}{\partial x\partial t^{2}}+E_{3}\frac{\partial^{2}}{\partial t\partial x}\right]h=\frac{1}{\text{K}\left(r+\text{K}\right)}\frac{\partial }{\partial r}\left[{\left(r+k\right)}^{2}S_{rx}\right]+\frac{H^{2}\psi_{r}}{\text{K}{\left(r+\text{K}\right)}^{2}}\;at\;r=\pm h,$$ (27)

where *ϵ (= a/d*_*1*_*)*, *δ (= d*_*1*_*/λ)* and K represent the dimensionless amplitude ratio, wave number and curvature parameter respectively. $E_{1}=-\frac{\tau d_{1}^{3}}{\lambda_{1}^{3}\mu c}$, ${\text{E}}_{2}=\frac{m_{1}cd_{1}^{3}}{\lambda_{1}^{3}\mu c}$, ${\text{E}}_{3}=\frac{dd_{1}^{3}}{\lambda_{1}^{2}\mu }$ the non-dimensional elasticity parameters, Re *(= cd*_*1*_ */ν)* depicts the Reynolds number, *Pr (= μC*_*p*_*/κ)* the Prandtl number, *We (= mc/d*_*1*_*)* the Weissenberg number, $H(=B_{0}d\sqrt{\sigma /\mu })$ the Hartman number, *Ec (= c*^*2*^*/C*_*p*_*T*_*0*_*)* the Eckert number, *Br (= EcPr)* the Brinkman number and *Bi*_*1*_ *(= h*_*1*_*/d)* the Biot number. Further the dimensionless form of extra stress tensor after invoking long wavelength and low Reynolds number approximation becomes

$$S_{rx}=\alpha \left(-\psi_{rr}+\frac{\psi_{r}}{\left(r+\text{K}\right)}\right)-\frac{\beta }{6}{\left(-\psi_{rr}+\frac{\psi_{r}}{\left(r+\text{K}\right)}\right)}^{3},$$ (28)

with $\alpha \;=\;\frac{A}{\mu C}$ and $\beta \;=\;\frac{\alpha c^{2}}{C^{2}d_{1}^{2}}$. Heat transfer coefficient is given by

$$Z=h_{x}\theta_{r}\left(h\right).$$

## Solution Methodology

The governing equations are highly non-linear and exact solution seems impossible. Therefore, perturbation method for small parameter *β* is used to find the solution. Thus we expand *ψ*, *S*_*rx*_, *θ* and *Z* as follows:

$$\psi =\psi_{0}+\beta \psi_{1}+\ldots \;,$$ (29)

$$S_{rx}=S_{0rx}+\beta S_{1rx}+\ldots \;,$$ (30)

$$\theta =\theta_{0}+\beta \theta_{1}+\ldots \;,$$ (31)

$$Z=Z_{0}+\beta Z_{1}+\ldots \;,$$ (32)

### Zeroth order system and solution

Using eqs (29)–(32) into eqs (21)–(28) and comparing the coefficients of *β*^*0*^ we have

$$\frac{\partial }{\partial r}\left[\frac{1}{\text{K}\left(r+\text{K}\right)}\frac{\partial }{\partial r}\left[{\left(r+\text{K}\right)}^{2}S_{0rx}\right]+\frac{H^{2}\psi_{0r}}{\text{K}\left(r+\text{K}\right)}\right]=0,$$ (33)

$$\left[\frac{\partial^{2}}{\partial r^{2}}+\frac{1}{\left(r+\text{K}\right)}\frac{\partial }{\partial r}\right]\theta_{0}=-Br\left[S_{0xr}\left(-\psi_{0rr}+\frac{\psi_{0r}}{\left(r+\text{K}\right)}\right)\right],$$ (34)

$$\psi_{0r}=0\;at\;r=\pm h\;,$$ (35)

$$\frac{\partial \theta_{0}}{\partial r}+Bi_{1}\theta_{0}=0\;at\;r=h,$$ (36)

$$\frac{\partial \theta_{0}}{\partial r}-Bi_{1}\theta_{0}=0\;at\;r=-h,$$ (37)

$$\left[E_{1}\frac{\partial^{3}h}{\partial x^{3}}+E_{2}\frac{\partial^{3}h}{\partial x\partial t^{2}}+E_{3}\frac{\partial^{2}h}{\partial t\partial x}\right]=\frac{1}{\text{K}\left(r+\text{K}\right)}\frac{\partial }{\partial r}\left[{\left(r+\text{K}\right)}^{2}S_{0rx}\right]+\frac{H^{2}\psi_{0r}}{\text{K}{\left(r+\text{K}\right)}^{2}}\;at\;r=\pm h,$$ (38)

with

$$S_{0rx}=\left(-\psi_{0rr}+\frac{\psi_{0r}}{\left(r+\text{K}\right)}\right).$$

Solving the above system we get

$$\psi_{0}=\frac{\sqrt{\alpha }{\left(r+\text{K}\right)}^{\frac{1+\sqrt{H^{2}+\alpha }}{\sqrt{\alpha }}}}{\sqrt{H^{2}+\alpha }+\sqrt{\alpha }}{\text{C}}_{1}-\frac{\sqrt{\alpha }{\left(r+\text{K}\right)}^{\frac{1-\sqrt{H^{2}+\alpha }}{\sqrt{\alpha }}}}{\sqrt{H^{2}+\alpha }-\sqrt{\alpha }}{\text{C}}_{2}+r\left(r+\text{K}\right)C_{3}+C_{4},$$ (39)

$$\theta_{0}=B_{2}+\frac{1}{4}[-Br{\left(r+\text{K}\right)}^{\frac{-2\sqrt{H^{2}+\alpha }}{\sqrt{\alpha }}}\alpha \left({\left(r+\text{K}\right)}^{\frac{4\sqrt{H^{2}+\alpha }}{\sqrt{\alpha }}}B_{15}+B_{16}\right)+4B_{1}\text{ln}\left(r+\text{K}\right)+4BrB_{17}H^{2}{\left(\text{ln}\left(r+\text{K}\right)\right)}^{2}]\;,$$ (40)

and heat transfer coefficient is given by

$$Z_{0}=h_{x}[2B_{1}+Br\left(h+\text{K}\right)\frac{-2\sqrt{H^{2}+\alpha }}{\sqrt{\alpha }}\left(B_{13}-B_{14}\left(\text{K}+h\right)\frac{4\sqrt{H^{2}+\alpha }}{\sqrt{\alpha }}\right)\sqrt{\alpha }\;\sqrt{H^{2}+\alpha }\;+8BrB_{17}H^{2}\text{ln}\left(\text{K}+h\right)/\left(\text{K}+h\right)].$$ (41)

### First order system and solution

$$\frac{\partial }{\partial r}\left[\frac{1}{\text{K}\left(r+\text{K}\right)}\frac{\partial }{\partial r}\left[{\left(r+\text{K}\right)}^{2}S_{1rx}\right]+\frac{H^{2}\psi_{1r}}{\text{K}\left(r+\text{K}\right)}\right]=0,$$ (42)

$$\left[\frac{\partial^{2}}{\partial r^{2}}+\frac{1}{\left(r+\text{K}\right)}\frac{\partial }{\partial r}\right]\theta_{1}=-Br\left[S_{1xr}\left(-\psi_{0rr}+\frac{\psi_{0r}}{\left(r+\text{K}\right)}\right)+S_{0xr}\left(-\psi_{1rr}+\frac{\psi_{1r}}{\left(r+\text{K}\right)}\right)\right],$$ (43)

$$\psi_{1r}=0\;at\;r=\pm h\;,$$ (44)

$$\frac{\partial \theta_{1}}{\partial r}+Bi_{1}\theta_{1}=0\;at\;r=h\;,$$ (45)

$$\frac{\partial \theta_{1}}{\partial r}+Bi_{1}\theta_{1}=0\;at\;r=-h\;,$$ (46)

$$\frac{1}{\left(r+\text{K}\right)}\frac{\partial }{\partial r}\left[{\left(r+\text{K}\right)}^{2}S_{1rx}\right]+\frac{H^{2}\psi_{1r}}{{\left(r+\text{K}\right)}^{2}}=0\;at\;r=\pm h,$$ (47)

with

$$S_{1rx}=\alpha \left(-\psi_{0rr}+\frac{\psi_{0r}}{r+\text{K}}\right)-\frac{\beta }{6}{\left(-\psi_{0rr}+\frac{\psi_{0r}}{\left(r+\text{K}\right)}\right)}^{3}\;.$$ (48)

The results corresponding to first order system are

$$\begin{array}{l} \psi_{1}=-C_{1}^{2}C_{2}H^{2}{\left(\text{K}+r\right)}^{\frac{-1+\sqrt{H^{2}+\alpha }}{\sqrt{\alpha }}}C_{9}+C_{2}^{2}C_{1}H^{2}{\left(\text{K}+r\right)}^{\frac{-1-\sqrt{H^{2}+\alpha }}{\sqrt{\alpha }}}C_{10}+C_{1}^{3}{\left(\text{K}+r\right)}^{\frac{-1+3\sqrt{H^{2}+\alpha }}{\sqrt{\alpha }}}\\ \;\times C_{11}-C_{2}^{3}{\left(\text{K}+r\right)}^{\frac{-1-3\sqrt{H^{2}+\alpha }}{\sqrt{\alpha }}}C_{12}+{\left(\text{K}+r\right)}^{\frac{\sqrt{H^{2}+\alpha }}{\sqrt{\alpha }}}\left(\text{K}\sqrt{\alpha }C_{13}+r\sqrt{\alpha }C_{14}\right)C_{5}+{\left(\text{K}+r\right)}^{\frac{-\sqrt{H^{2}+\alpha }}{\sqrt{\alpha }}}\\ \;\times \left(\text{K}\sqrt{\alpha }C_{15}+r\sqrt{\alpha }C_{16}\right)C_{6}+\text{K}rC_{7}+\frac{1}{2r^{2}C_{7}}+C_{8}, \end{array}$$ (49)

$$\begin{array}{l} \theta_{1}=1/96[\frac{6BrC_{1}^{2}C_{2}^{2}H^{4}}{{\left(\text{K}+r\right)}^{2}\alpha^{2}}+BrC_{1}^{4}H^{2}{\left(\text{K}+r\right)}^{\frac{-2+4\sqrt{H^{2}+\alpha }}{\sqrt{\alpha }}}B_{7}-BrC_{2}^{4}H^{2}{\left(\text{K}+r\right)}^{\frac{-2-4\sqrt{H^{2}+\alpha }}{\sqrt{\alpha }}}B_{8}\\ \;+4{\left(\text{K}+r\right)}^{\frac{2\sqrt{H^{2}+\alpha }}{\sqrt{\alpha }}}\left(B_{9}+\frac{B_{10}}{{\left(\text{K}+r\right)}^{2}}\right)+4{\left(\text{K}+r\right)}^{\frac{-2\sqrt{H^{2}+\alpha }}{\sqrt{\alpha }}}\left(B_{11}+\frac{B_{12}}{{\left(\text{K}+r\right)}^{2}}\right)+96B_{4}\\ \;96B_{3}\text{log}\left(\text{K}+r\right)+48Br\left(C_{2}C_{5}+C_{1}\left(2C_{2}+C_{6}\right)\right)H^{2}\text{log}{\left(\text{K}+r\right)}^{2}], \end{array}$$ (50)

and heat transfer coefficient is given by

$$\begin{array}{l} Z_{1}=\frac{1}{96{\left(\text{K}+h\right)}^{3}}h_{x}[(96B_{3}{\left(\text{K}+h\right)}^{2}-8B_{12}{\left(\text{K}+h\right)}^{\frac{-2\sqrt{H^{2}+\alpha }}{\sqrt{\alpha }}}-8B_{10}{\left(\text{K}+h\right)}^{\frac{2\sqrt{H^{2}+\alpha }}{\sqrt{\alpha }}}-\frac{BrC_{1}^{2}}{\alpha^{2}}C_{2}^{2}H^{4}\\ \;+\frac{1}{\sqrt{\alpha }}\{-8{\left(\text{K}+h\right)}^{\frac{-2\sqrt{H^{2}+\alpha }}{\sqrt{\alpha }}}\left(B_{11}{\left(\text{K}+h\right)}^{2}+B_{12}\right)\sqrt{H^{2}+\alpha }\\ \;+8{\left(\text{K}+h\right)}^{\frac{2\sqrt{H^{2}+\alpha }}{\sqrt{\alpha }}}\left(B_{9}{\left(\text{K}+h\right)}^{2}+B_{10}\right)\sqrt{H^{2}+\alpha }\}\\ \;-BrC_{2}^{4}H^{2}{\left(\text{K}+h\right)}^{\frac{-4\sqrt{H^{2}+\alpha }}{\sqrt{\alpha }}}B_{8}\left(-2-\frac{4\sqrt{H^{2}+\alpha }}{\sqrt{\alpha }}\right)\\ \;+96Br\left(C_{2}C_{5}+C_{1}\left(2C_{2}+C_{6}\right)\right)H^{2}{\left(\text{K}+h\right)}^{2}\text{log}\left(\text{K}+h\right)\\ \;+BrC_{1}^{4}H^{2}B_{7}{\left(\text{K}+h\right)}^{\frac{4\sqrt{H^{2}+\alpha }}{\sqrt{\alpha }}}B_{8}\left(-2+\frac{4\sqrt{H^{2}+\alpha }}{\sqrt{\alpha }}\right)]. \end{array}$$ (51)

Here the algebraic values of C_1_ → C_6_ and B_1_ → B_12_ can be evaluated using MATHEMATICA.

## Results and Discussion

This portion analyzes the impact of several parameters of interest on the velocity (*u)*, temperature distribution (*θ)*, heat transfer coefficient (*Z)* and stream function (*ψ)*.

**Fig 2:**
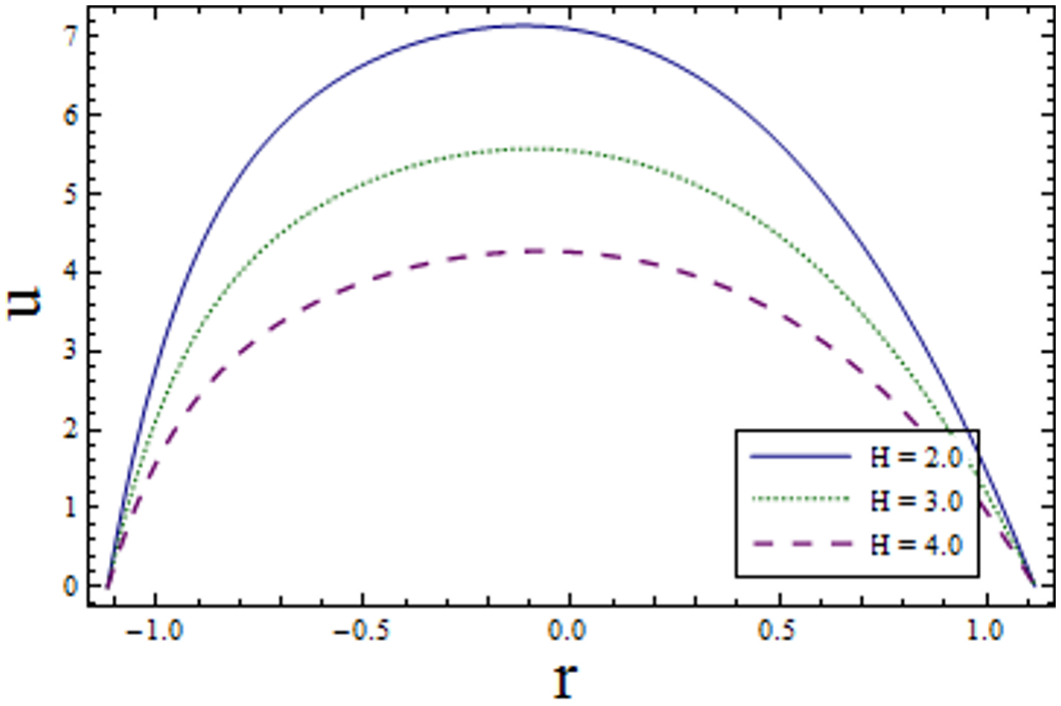
Variation of *H* on *u* when ϵ = 0.2, x = 0.2, t = 0.1, E_1_ = 0.2, E_2_ = 0.01, E_3_ = 0.1, k = 3.5, α = 1.5 and β = 0.2.

**Fig 3:**
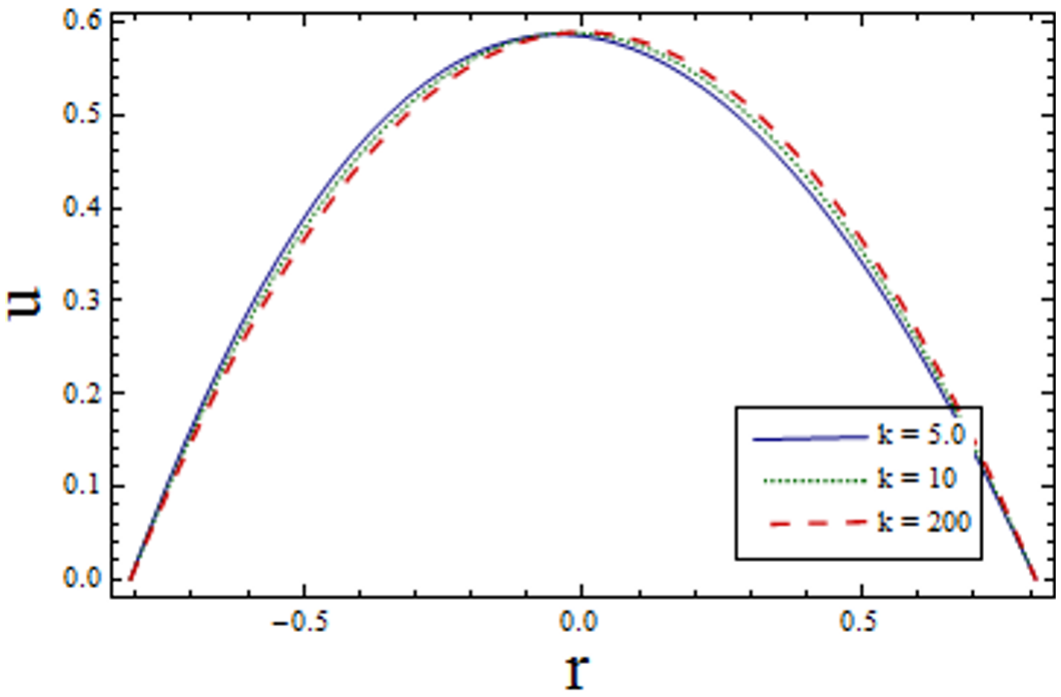
Variation of *k* on *u* when ϵ = 0.2, x = 0.2, t = 0.1, E_1_ = 0.02, E_2_ = 0.01, E_3_ = 0.3, H = 0.2, α = 1.5 and β = 0.2.

**Fig 4:**
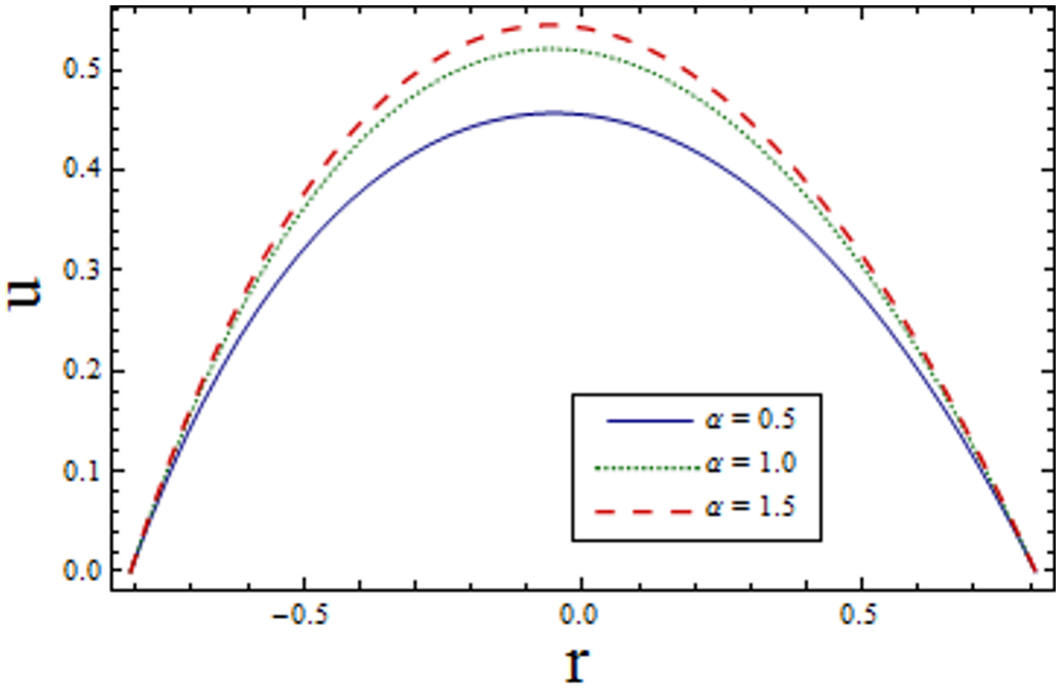
Variation of *α* on *u* when ϵ = 0.2, x = 0.2, t = 0.1, E_1_ = 0.04, E_2_ = 0.03, E_3_ = 0.1, k = 3.5, H = 2.5 and β = 0.2.

**Fig 5:**
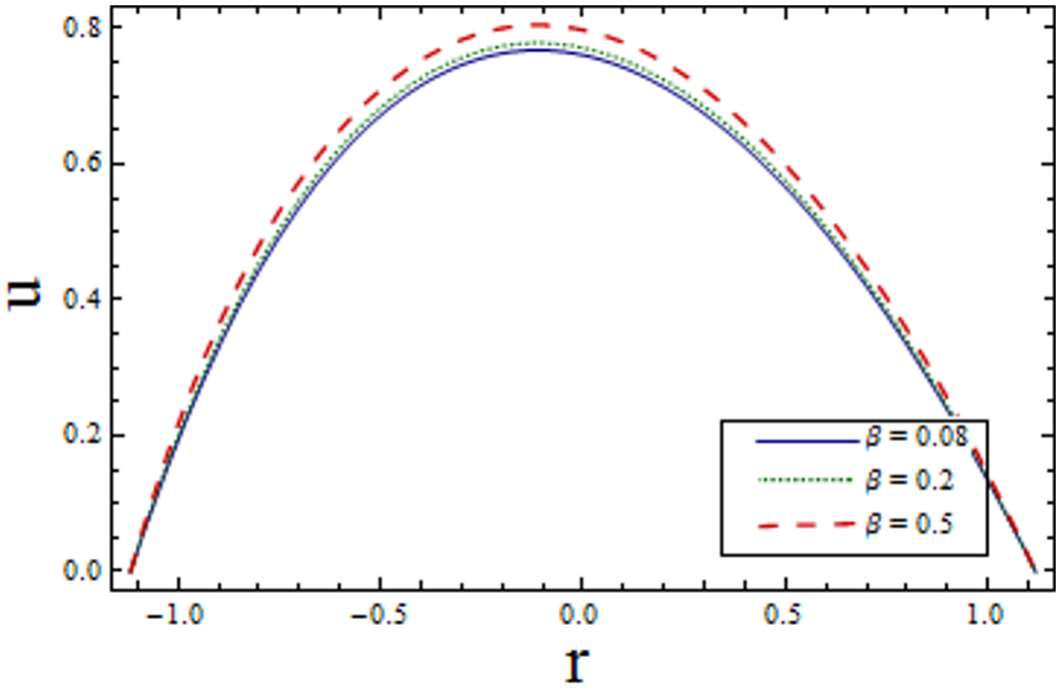
Variation of *β* on *u* when ϵ = 0.2, x = 0.2, t = 0.1, E_1_ = 0.04, E_2_ = 0.03, E_3_ = 0.3, k = 3.5, α = 1.5 and H = 2.0.

**Fig 6:**
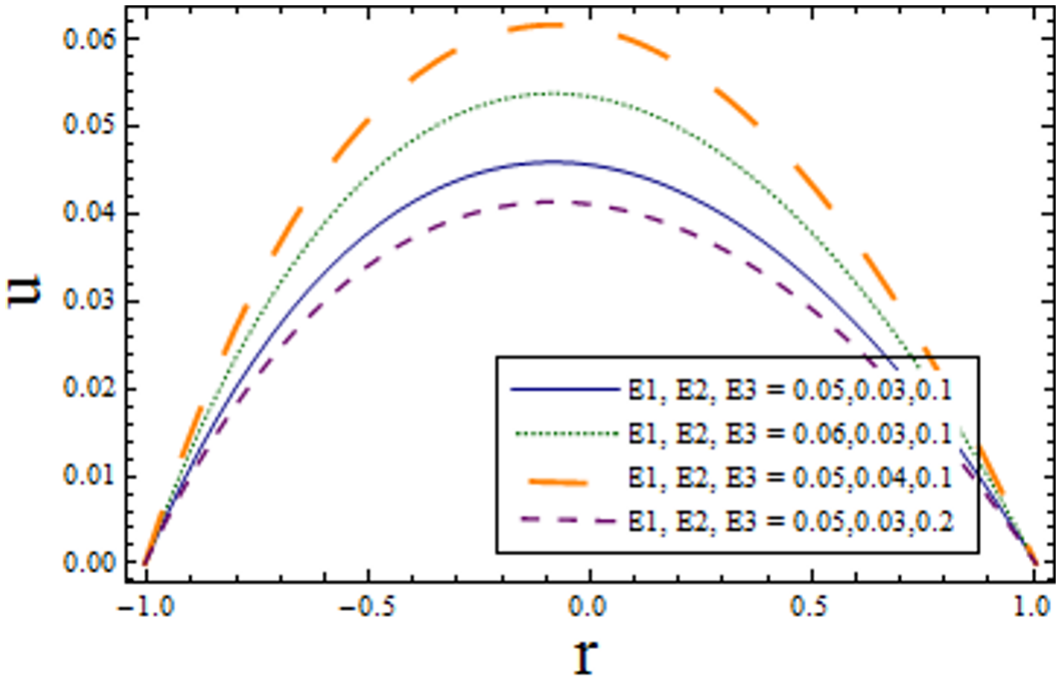
Variation of *complaint wall parameters* on *u* when ϵ = 0.2, x = 0.2, t = 0.1, k = 3.5, α = 1.5, H = 2.8 and β = 0.2.

Figs 2–6 are prepared to study velocity profile. It is shown by these figures that velocity profile is parabolic in nature. Also, maximum value is observed at the center of channel. Fig 2 shows decrease in *u* by enhancing *H*. This is due to the fact that when the magnetic field is applied in the transverse direction, it provides resistance to the flow which in turn decreases the velocity. Fig 3 shows increase in the axial velocity *u* increases for larger curvature *k* near upper half of the channel. Whereas opposite behavior is seen near lower wall. Figs 4 and 5 indicate that the axial velocity acts like an increasing function of Prandtl Eyring fluid parameters *α* and *β*. Fig 6 illustrates that with an increase in *E*_*1*_ and *E*_*2*_ the velocity enhances. It is due to the fact that less resistance is offered to the flow because of the wall elastance and thus velocity increases. However reverse effect is observed for *E*_*3*_. This is because of the fact that larger *E*_*3*_ more resistive force due to damping and thus velocity decreases. Here we observe that the results obtained are in good agreement with the one get by Hina et al. [27].

**Fig 7:**
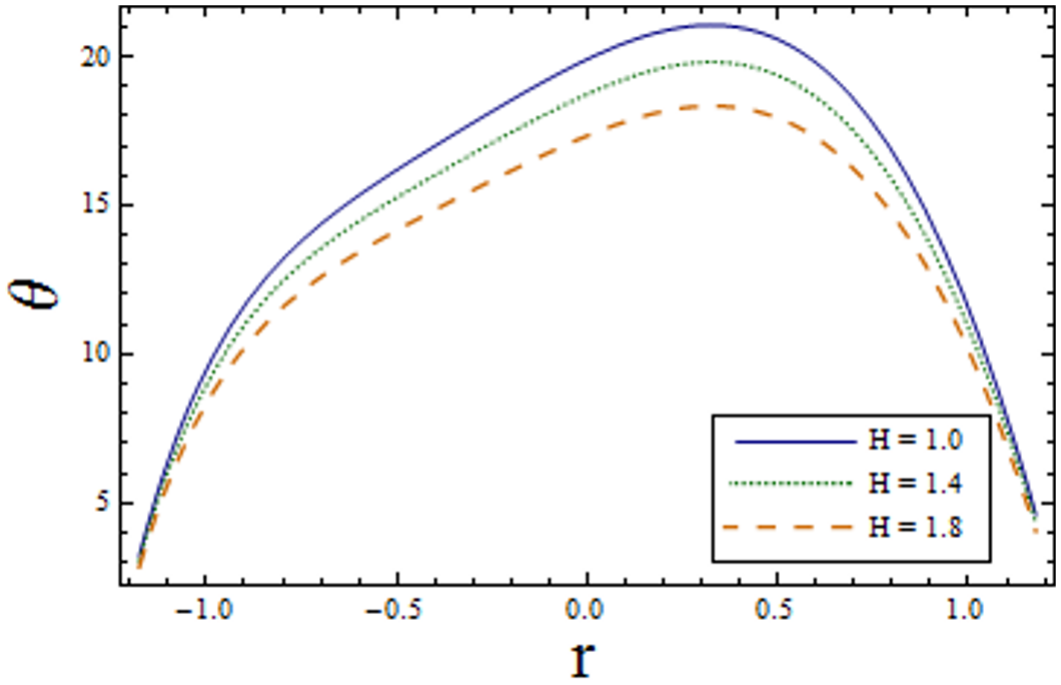
Variation of *H* on *θ* when ϵ = 0.2, x = 0.3, t = 0.1, Br = 2, E_1_ = 0.04, E_2_ = 0.03, E_3_ = 0.02, α = 1.5, β = 0.7, Bi_1_ = 10 and k = 4.

**Fig 8:**
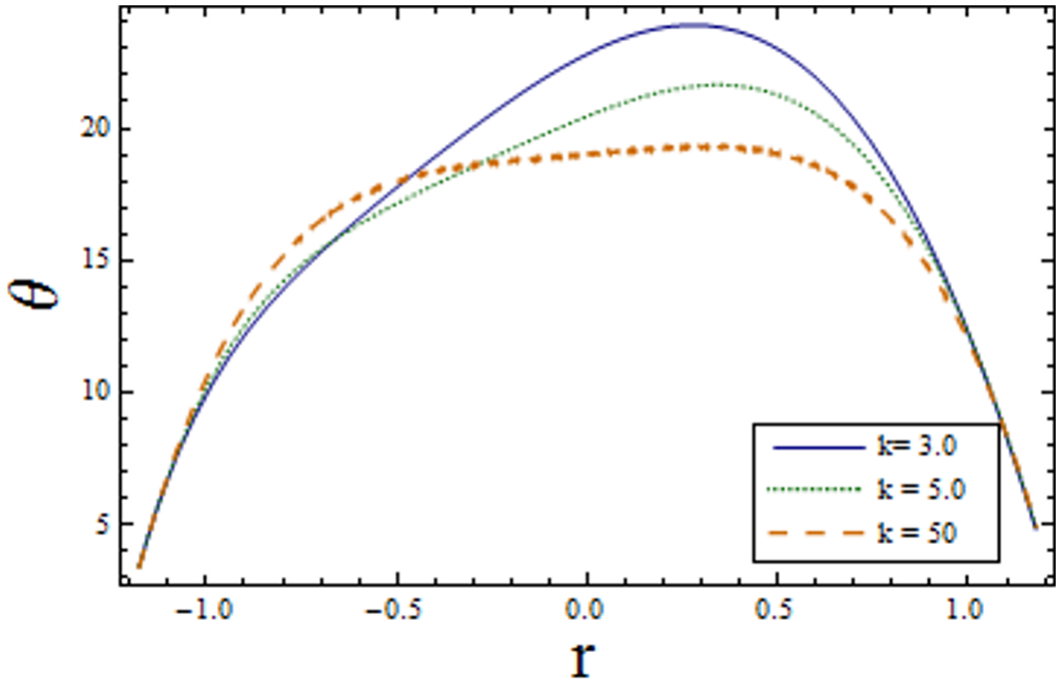
Variation of *k* on *θ* when ϵ = 0.2, x = 0.3, t = 0.1, Br = 2, E_1_ = 0.04, E_2_ = 0.03, E_3_ = 0.02, α = 1.5, β = 0.7, Bi_1_ = 10 and k = 0.2.

**Fig 9:**
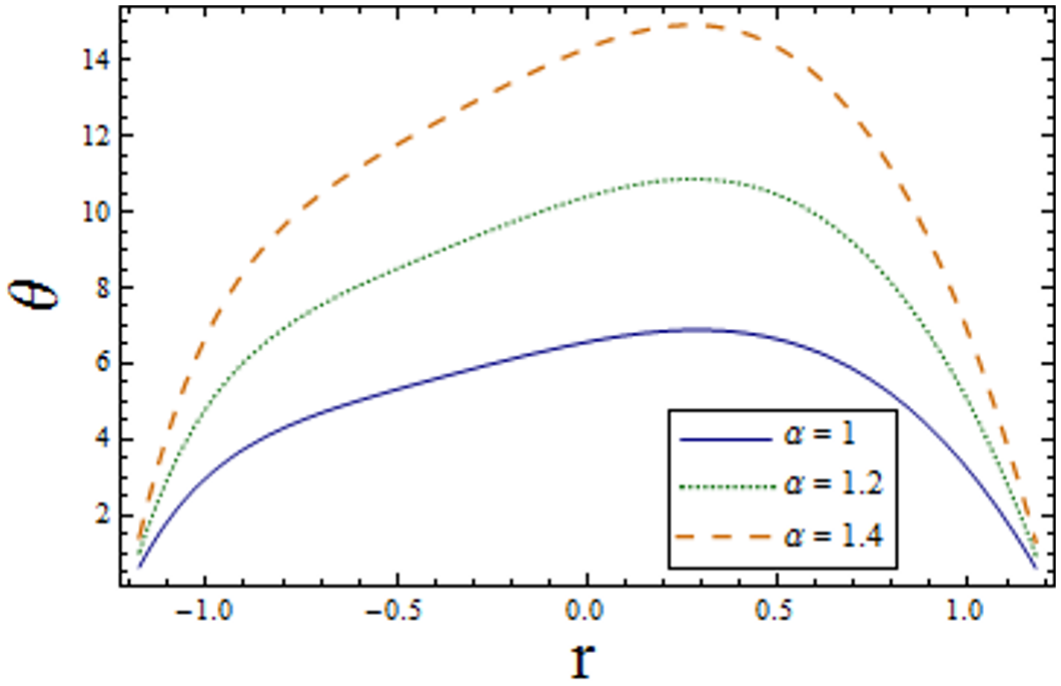
Variation of *α* on *θ* when ϵ = 0.2, x = 0.3, t = 0.1, Br = 2, E_1_ = 0.04, E_2_ = 0.03, E_3_ = 0.01, β = 0.7, H = 2.0, Bi_1_ = 10 and k = 3.5.

**Fig 10:**
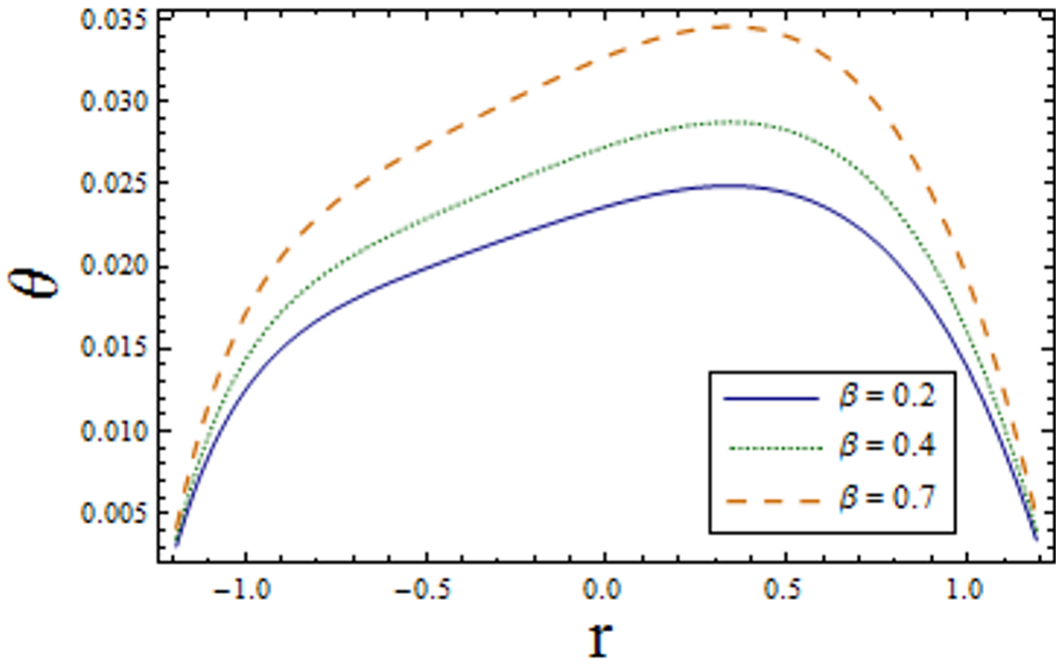
Variation of *β* on *θ* when ϵ = 0.2, x = 0.3, t = 0.1, Br = 2, E_1_ = 0.04, E_2_ = 0.03, E_3_ = 0.01, α = 1.5, H = 2.0, Bi_1_ = 10 and k = 3.5.

**Fig 11:**
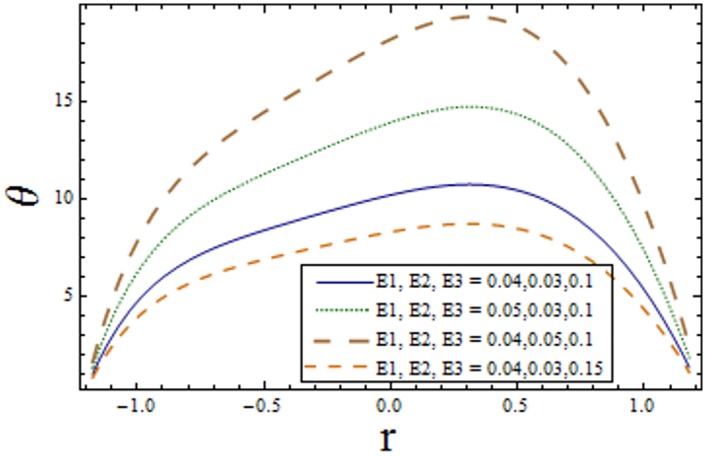
Variation of *complaint wall parameters* on *θ* when ϵ = 0.2, x = 0.3, t = 0.1, Br = 2, β = 0.7, α = 1.5, H = 2.0, Bi_1_ = 10 and k = 3.5.

**Fig 12:**
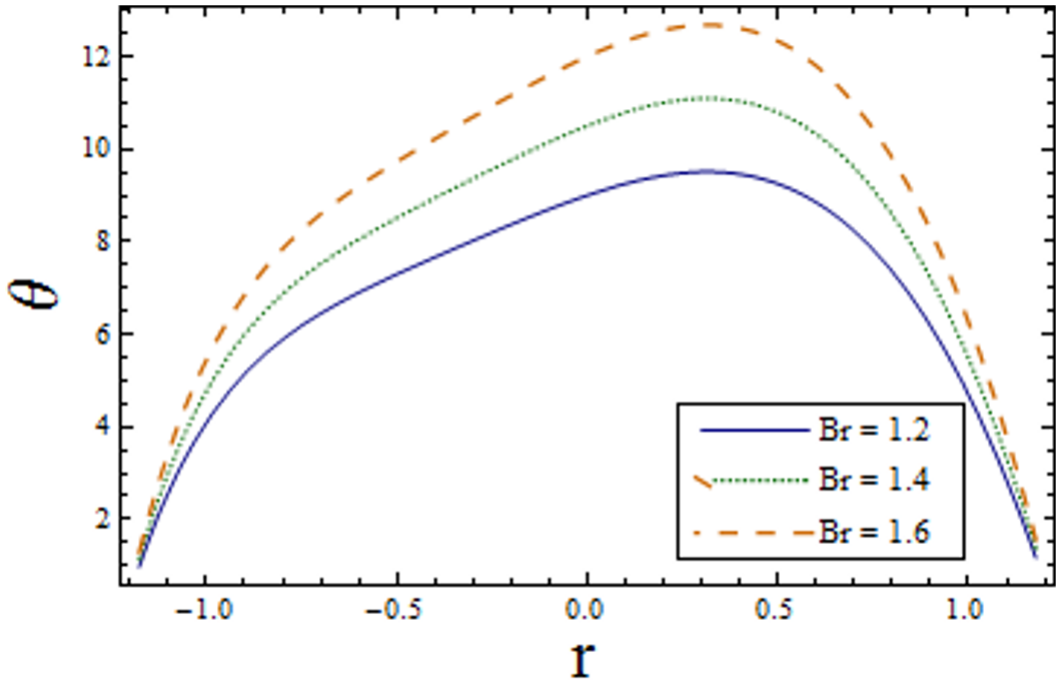
Variation of *Br* on *θ* when ϵ = 0.2, x = 0.3, t = 0.1, β = 0.7, E_1_ = 0.04, E_2_ = 0.03, E_1_ = 0.01, α = 1.5, H = 2.0, Bi_1_ = 10 and k = 3.5.

**Fig 13:**
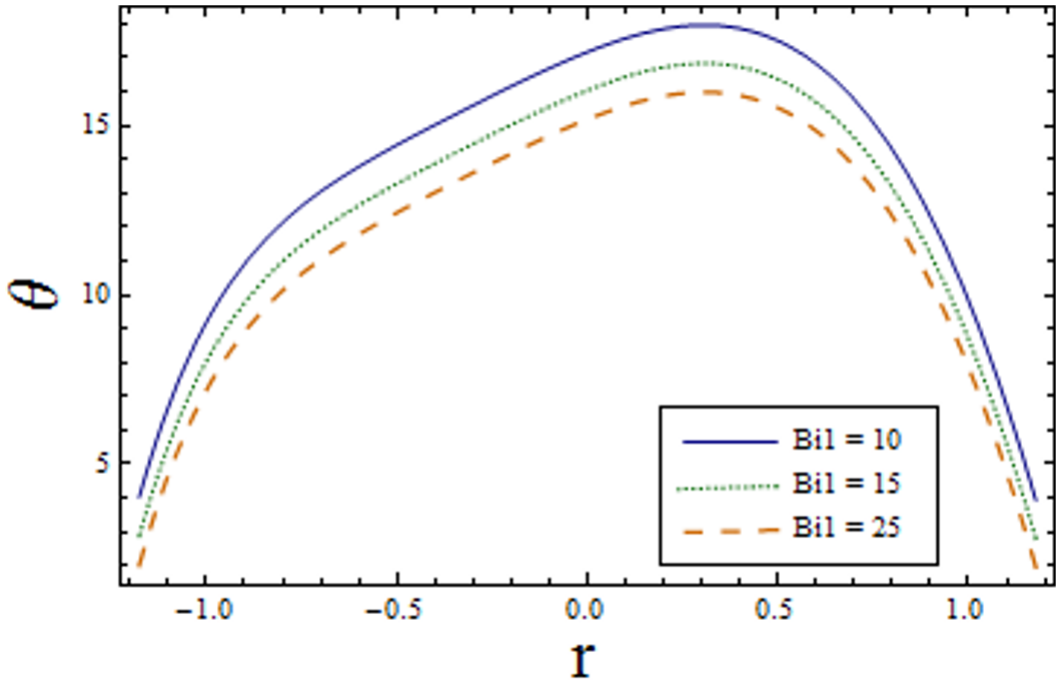
Variation of *Bi*_*1*_ on *θ* when ϵ = 0.2, x = 0.3, t = 0.1, E_1_ = 0.04, E_2_ = 0.03, E_3_ = 0.02, α = 1.5, H = 2.0, β = 0.7 and k = 3.5.

Figs 7–13 indicate the effect of significant parameters involved in the temperature distribution *θ*. Fig 7 reveals that *θ* decreases when Hartman number *H* is increased. Fig 8 shows that increasing curvature K of the channel, we get opposite results for *θ* in upper/lower half of the channel. Figs 9 and 10 show that the temperature profile increases for larger Prandtl Eyring fluid parameters *α* and *β*. It is shown in Fig 11 that temperature increases via *E*_*1*_ and *E*_*2*_ and it decreases through *E*_*3*_. Fig 12 illustrate that the temperature enhances when Brinkman number *Br* is increased. Fig 13 discloses that by increasing the *Bi*_*1*_ the temperature decreases. Here we have considered the values of Biot number much larger than 0.1 due to non-uniform temperature fields within the fluid. Temperature is the average kinetic energy of the molecules. Increase/decrease in temperature directly effects the velocity. Therefore, we get almost similar qualitative behavior for velocity and temperature profiles.

**Fig 14:**
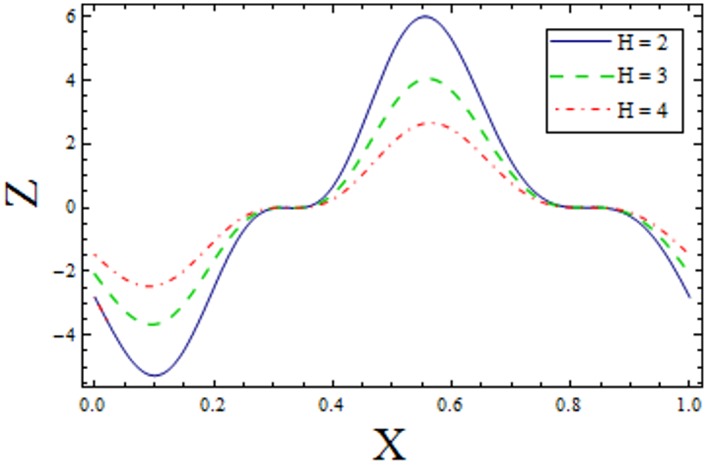
Variation of *H* on *Z* when ϵ = 0.2, t = 0.1, Br = 2, E_1_ = 0.04, E_2_ = 0.03, E_3_ = 0.02, α = 1.5, β = 0.7, Bi_1_ = 10 and k = 4.

**Fig 15:**
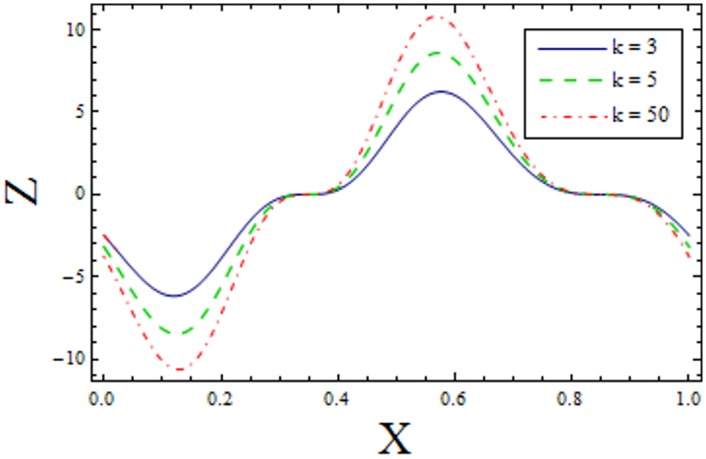
Variation of *k* on *Z* when ϵ = 0.2, t = 0.1, Br = 2, E_1_ = 0.04, E_2_ = 0.03, E_3_ = 0.1, α = 1.5, β = 0.7, Bi_1_ = 5, H = 2.

**Fig 16:**
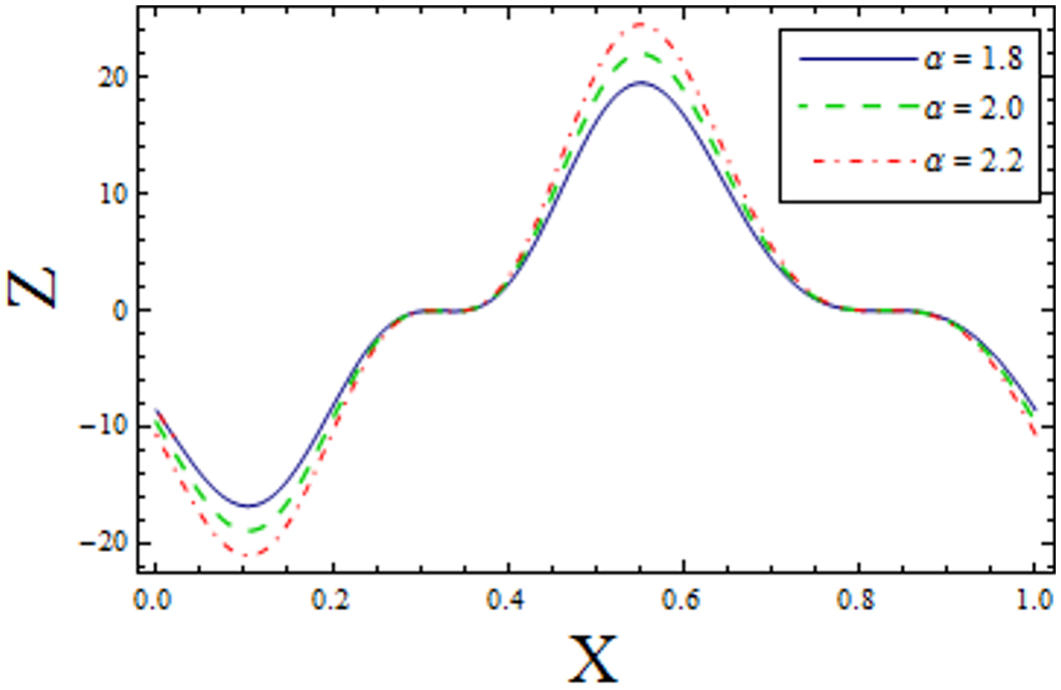
Variation of *α* on *Z* when ϵ = 0.2, t = 0.1, Br = 2, E_1_ = 0.04, E_2_ = 0.03, E_3_ = 0.1, H = 1.5, β = 0.7, Bi_1_ = 5 and k = 3.5.

**Fig 17:**
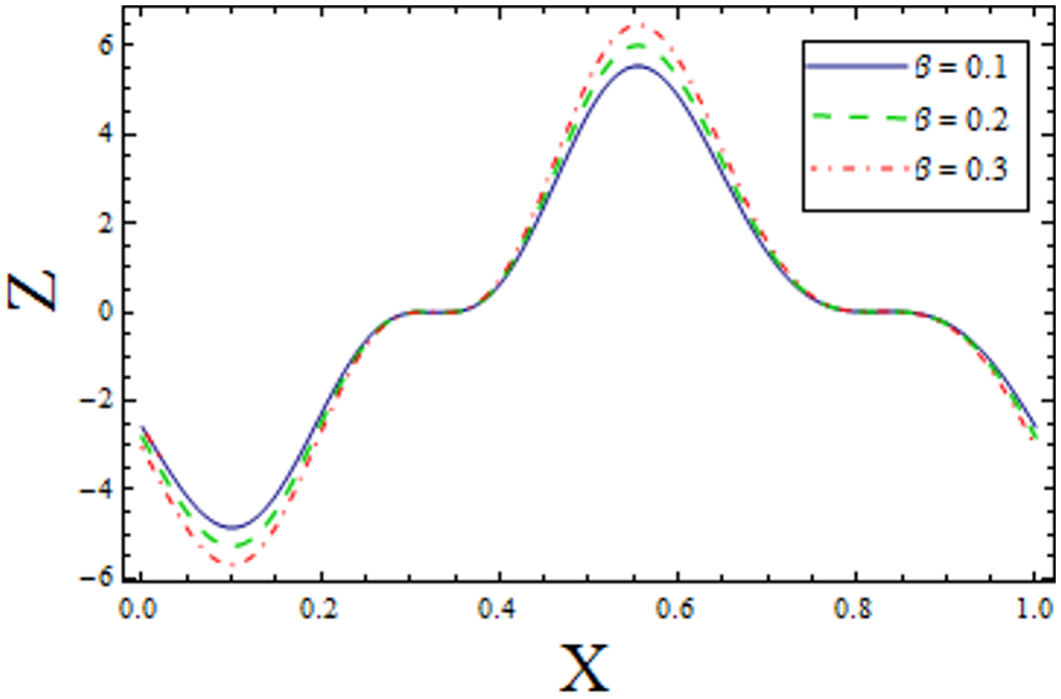
Variation of *β* on *Z* when ϵ = 0.2, t = 0.1, Br = 2, E_1_ = 0.04, E_2_ = 0.03, E_3_ = 0.1, α = 1.5, H = 2, Bi_1_ = 5 and k = 3.5.

**Fig 18:**
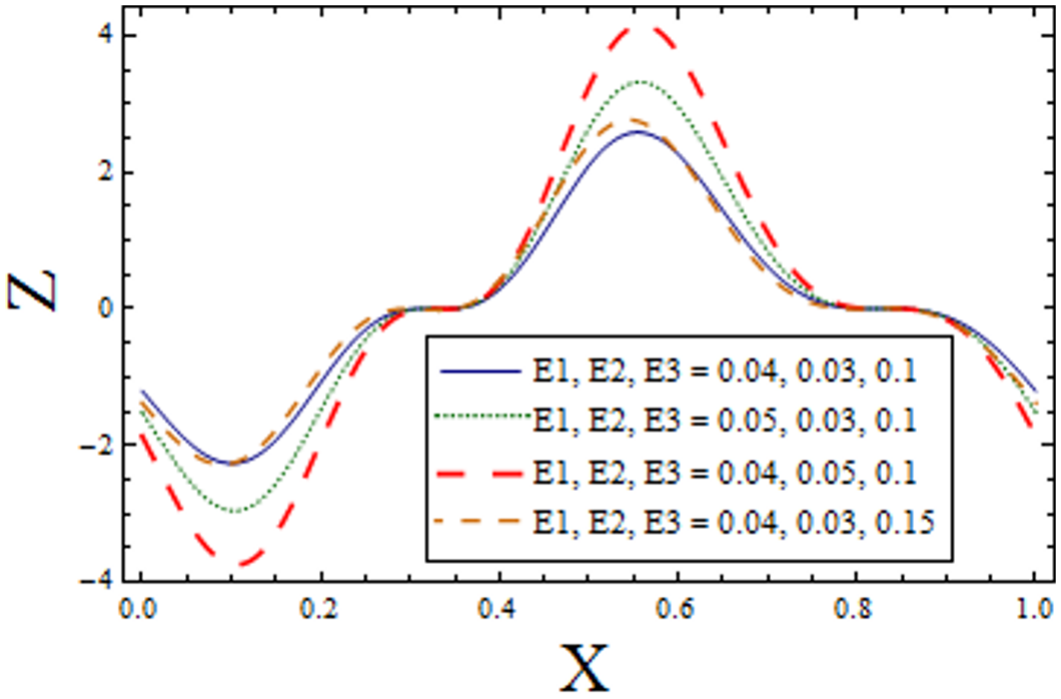
Variation of *complaint wall parameters* on *Z* when ϵ = 0.2, t = 0.1, Br = 2, H = 02, α = 1.5, β = 0.7, Bi_1_ = 10 and k = 0.5.

**Fig 19:**
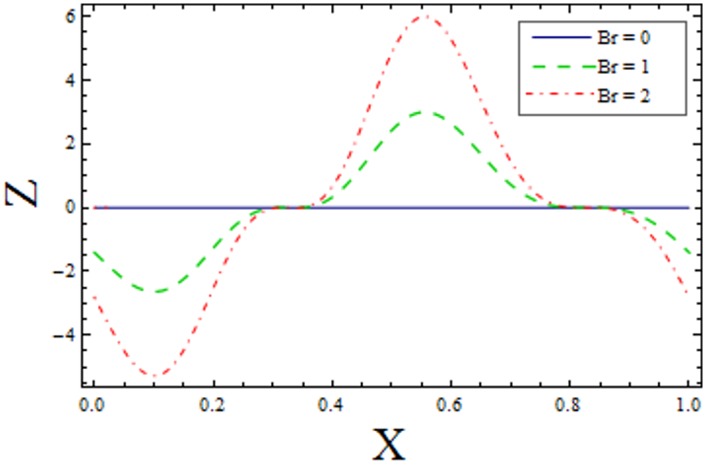
Variation of *Br* on *Z* when ϵ = 0.2, t = 0.1, H = 2, E_1_ = 0.04, E_2_ = 0.03, E_3_ = 0.1, α = 1.5, β = 0.7, Bi_1_ = 10 and k = 3.5.

**Fig 20:**
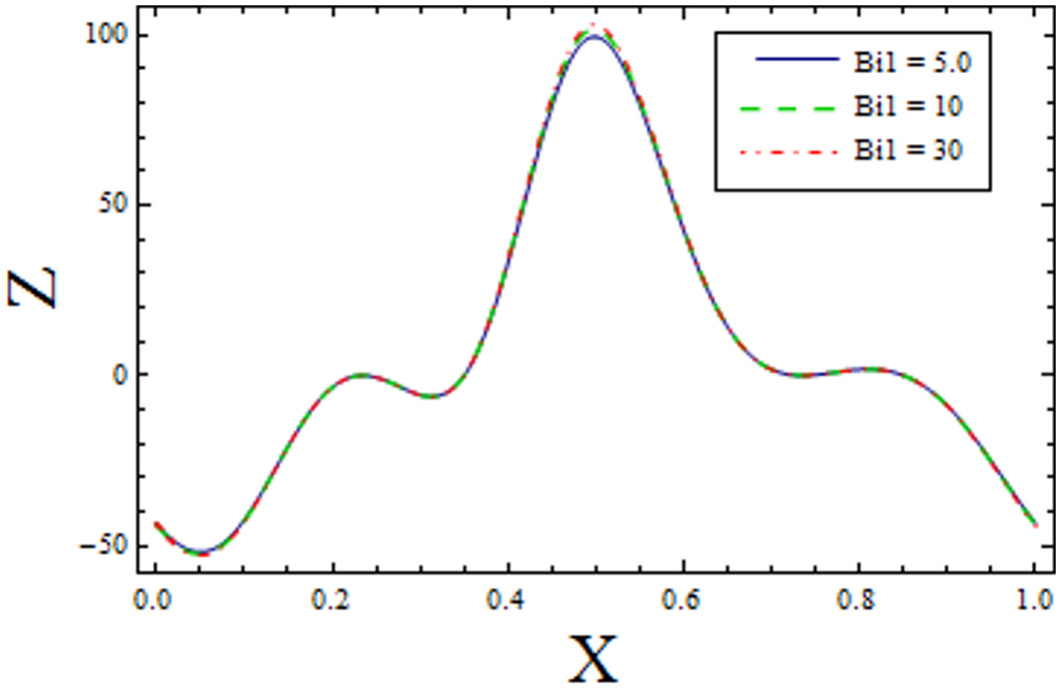
Variation of *Bi*_*1*_ on *Z* when ϵ = 0.2, t = 0.1, H = 2, E_1_ = 0.04, E_2_ = 0.03, E_3_ = 0.1, α = 1.5, β = 0.7, Br = 2, H = 2 and k = 3.5.

In Figs 14–20 show the impact of various values of emerging parameters of Z(x). Fig 14 portrays that magnitude of Z(x) decreases when Hartman number *H* is increased. Fig 15 shows that the Z(x) increases when curvature parameter K is increased. The magnitude of Z(x) increases for larger Prandtl Eyring fluid parameters *α* and *β* (Figs 16 and 17). Fig 18 depicts that absolute value of Z(x) increases when there is an increase in *E*_*1*_ and *E*_*2*_. However heat transfer coefficient decreases for *E*_*3*_. Fig 19 illustrates that absolute value of heat transfer coefficient enhances by increasing *Br*. Further *Z(x)* is increasing function of *Bi*_*1*_ (Fig 20).

**Fig 21:**
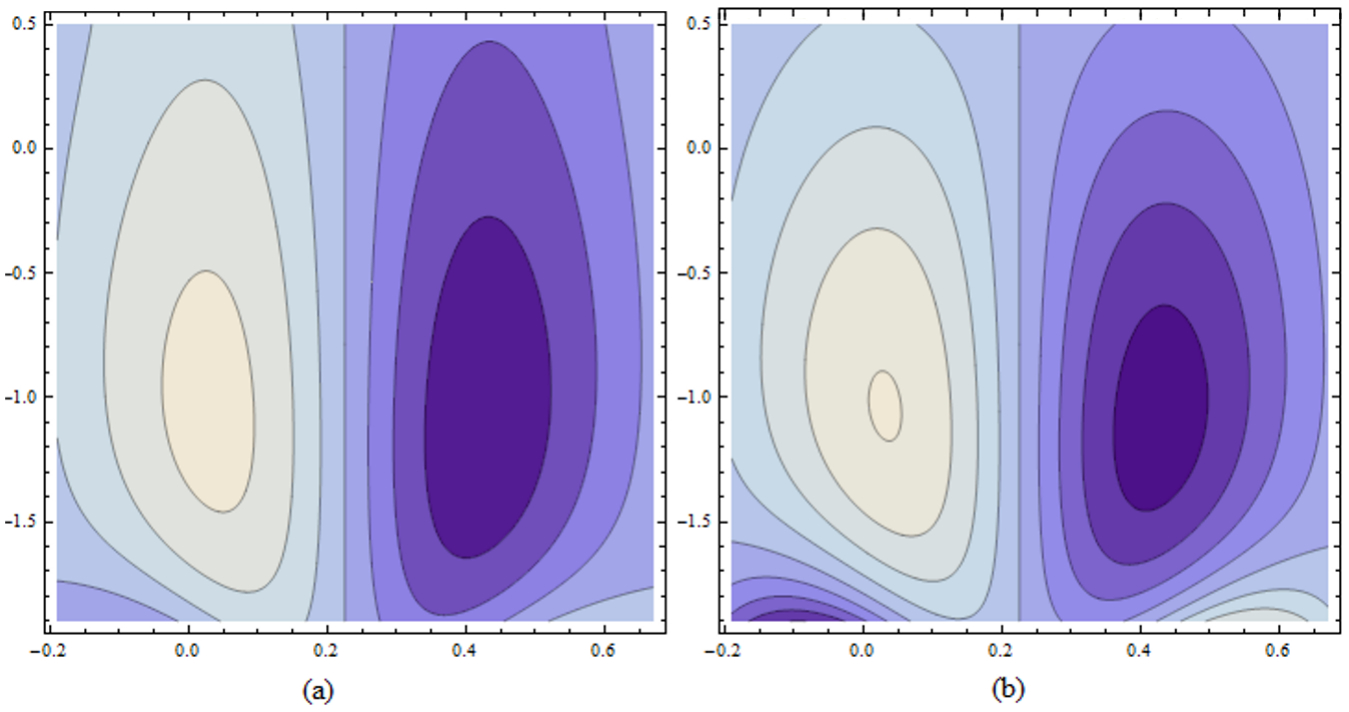
Variation of *H* on *ψ* for E_1_ = 0.02, E_2_ = 0.01, E_3_ = 0.03, α = 0.2, β = 0.02, ϵ = 0.2, t = 0.0, k = 3.5 when (a): H = 0.8 and (b): H = 1.1.

**Fig 22:**
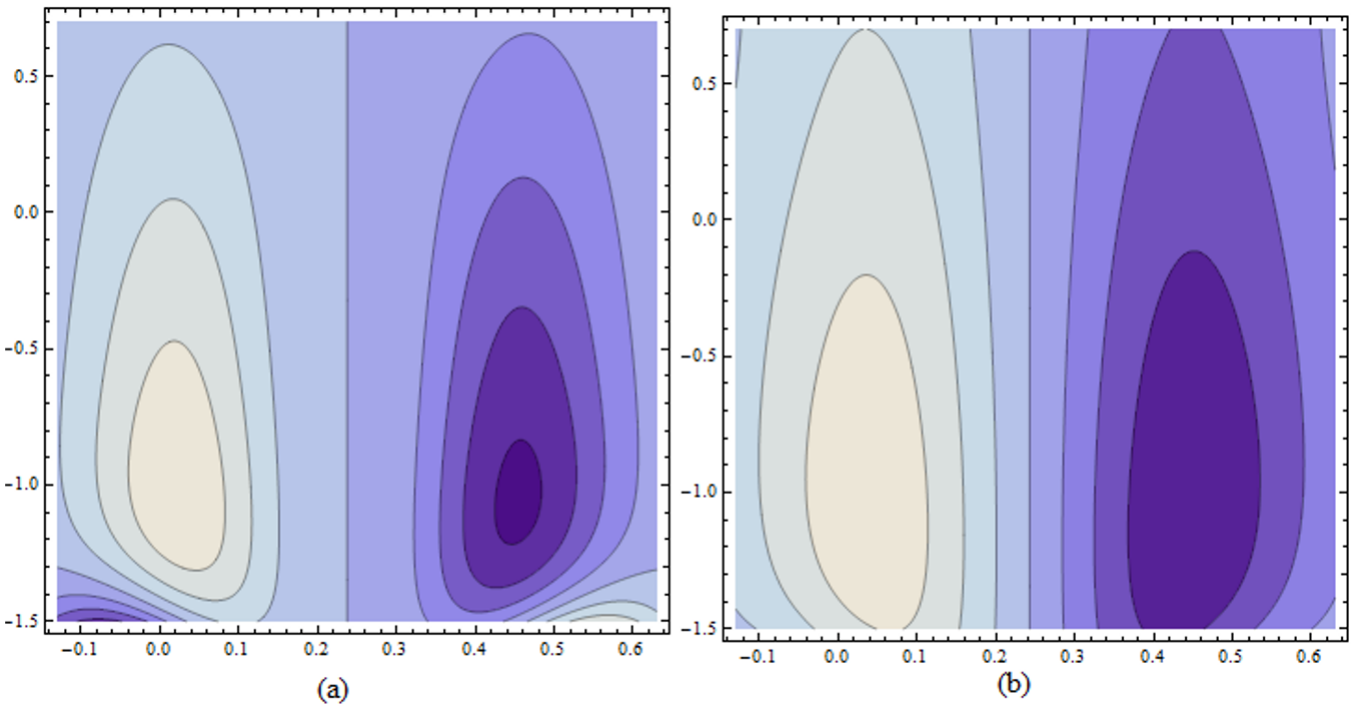
Variation of *k* on *ψ* for E_1_ = 0.02, E_2_ = 0.15, E_3_ = 0.05, α = 0.2, β = 0.02, ϵ = 0.2, t = 0.0, H = 0.8 when (a): k = 3.8 and (b): k = 5.

**Fig 23:**
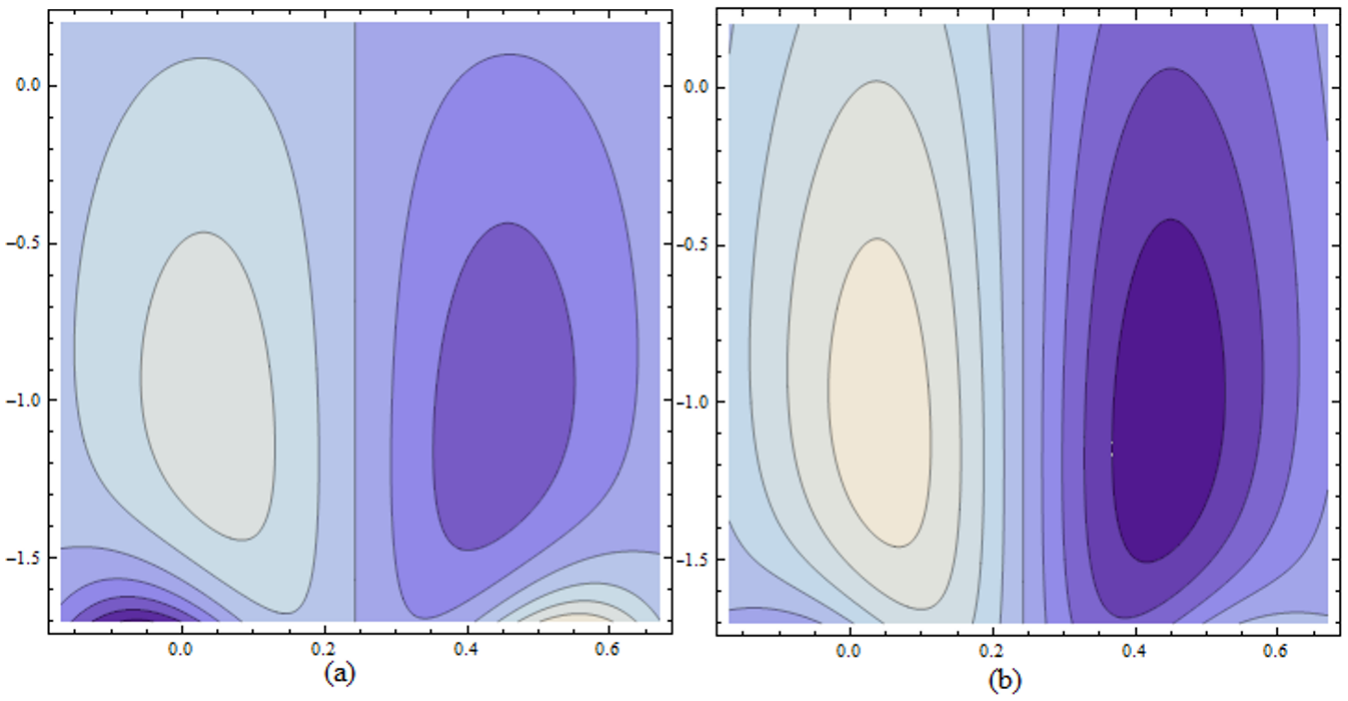
Variation of *α* on *ψ* for E_1_ = 0.02, E_2_ = 0.15, E_3_ = 0.05, k = 3.5, β = 0.02, ϵ = 0.2, t = 0.0, H = 2.0 when (a): α = 0.5 and (b): α = 1.1.

**Fig 24:**
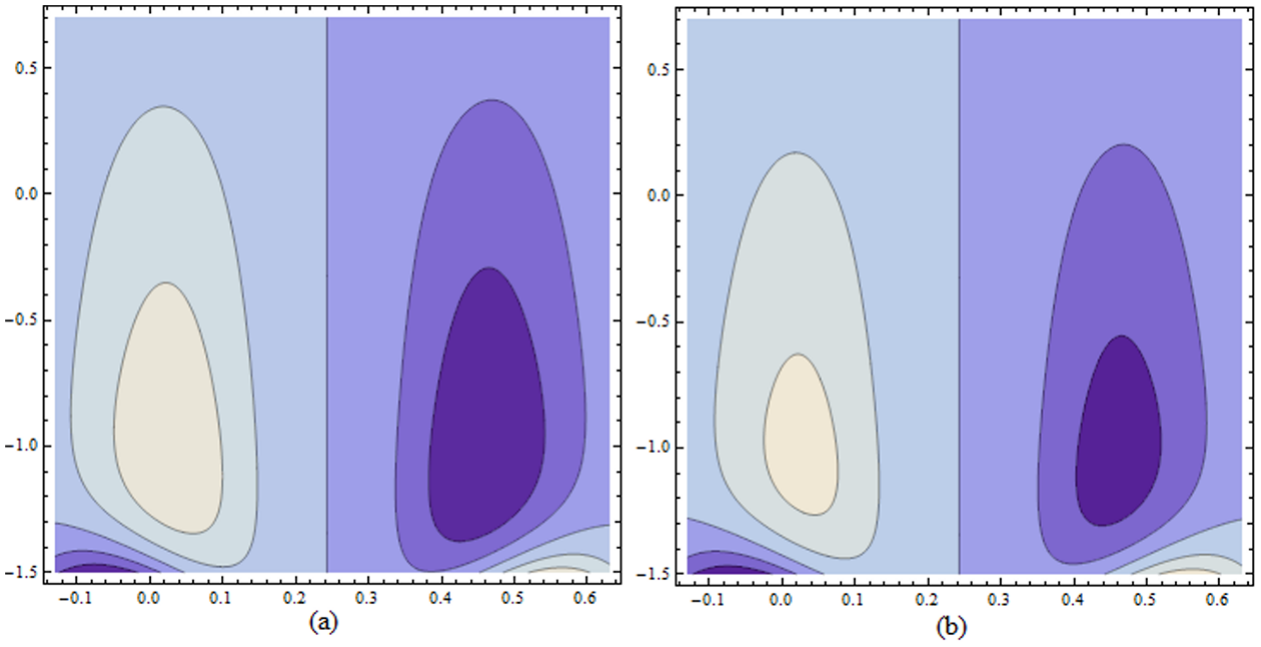
Variation of *β* on *ψ* for E_1_ = 0.02, E_2_ = 0.15, E_3_ = 0.05, α = 0.7, k = 3.5, ϵ = 0.2, t = 0.0, H = 2.0 when (a): β = 0.3 and (b): β = 1.5.

**Fig 25:**
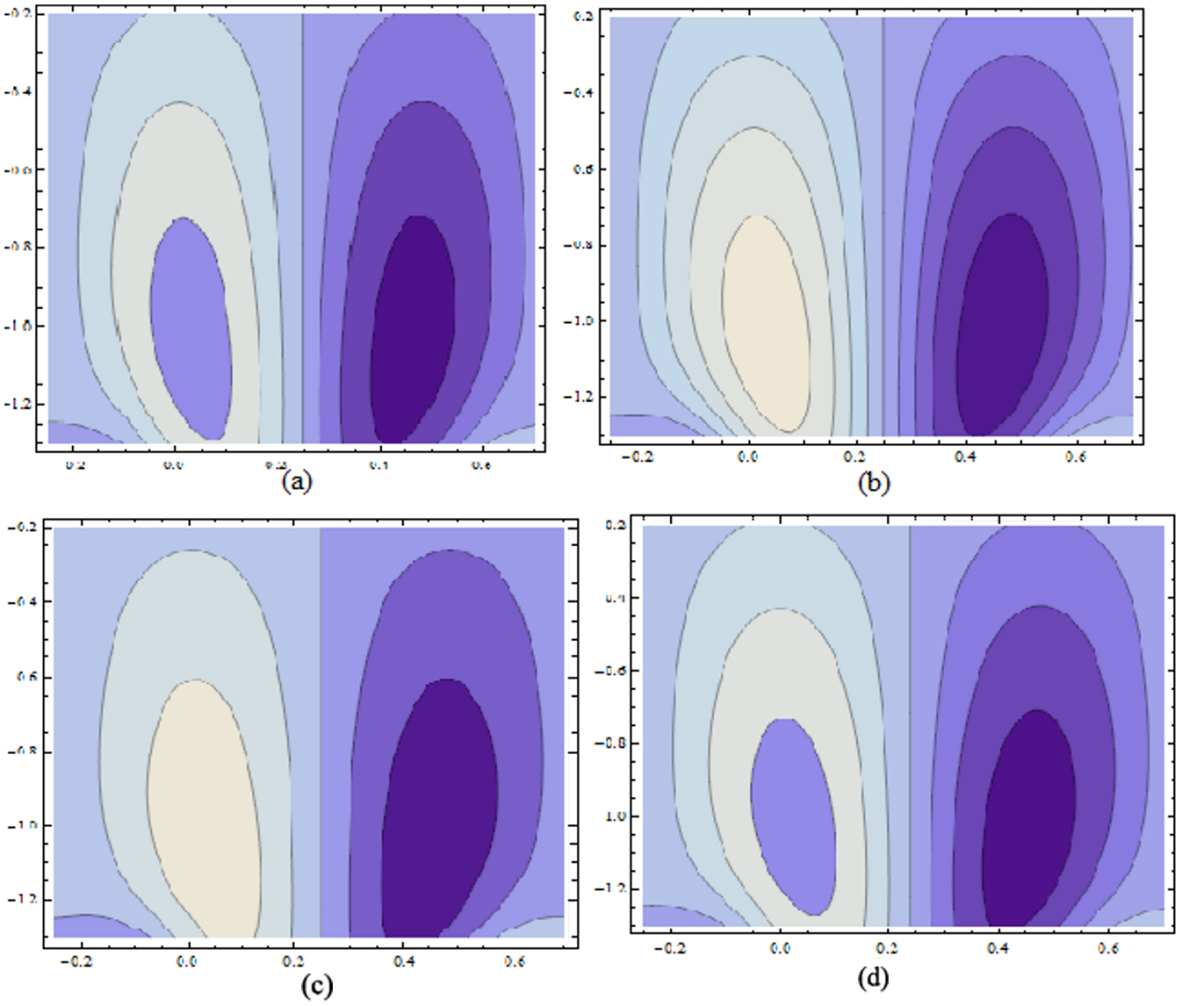
Variation of *wall properties* on *ψ* for H = 5, α = 1.5, k = 3.5, ϵ = 0.2, t = 0.0, β = 0.4 when (a): E_1_ = 0.03, E_2_ = 0.02, E_3_ = 0.1 (b): E_1_ = 0.04, E_2_ = 0.02, E_3_ = 0.1 (c): E_1_ = 0.03, E_2_ = 0.04, E_3_ = 0.1 and (d): E_1_ = 0.03, E_2_ = 0.02, E_3_ = 0.2.

Figs (21–25) display the streamline pattern for various values of invoked parameters. Fig 21a and 21b discusses the impact of Hartman number *H* on streamlines. Decrease in the size is noticed for increased *H*. Fig 22a and 22b show the effect of curvature parameter *k* on the streamlines. These figures show that the bolus size enhances for larger K. Fig 23a and 23b illustrate the fact that the trapped bolus size increases when fluid parameter *α* is enhanced. Number of streamlines are more. Fig 24a and 24b show that size of trapped bolus decreases when we increase the values of fluid parameter *β*. We have analyzed through Fig 25a–25d that the streamlines increases through the increase in elastic parameters *E*_*1*_ and *E*_*2*_ while increase in *E*_*3*_ has no show significant effect. Moreover, by taking *α = β = H = Bi*_*1*_ *= 0* results can be obtained for viscous case [24].

## Conclusions

Peristaltic motion of MHD Prandtl Eyring fluid flowing through curved geometry is discussed. Walls of channel are chosen to be compliant. Heat transfer phenomenon is also analyzed. Main findings of this study are:

1. Symmetry of velocity profile about the centre line is disturbed for the flow in curved channel.
2. The velocity profile has decreasing behavior for increasing values of Hartman number *H*.
3. Temperature profile is a decreasing function of Biot number *Bi*_*1*_.
4. The absolute value of heat transfer coefficient for planer channel is higher than the curved one.
5. Size of trapped bolus increases for α but it decreases via *β*.
